# Temperature-Dependent Absorption of Ternary HfS_2−x_Se_x_ 2D Layered Semiconductors

**DOI:** 10.3390/ma15186304

**Published:** 2022-09-11

**Authors:** Der-Yuh Lin, Hung-Pin Hsu, Cheng-Wen Wang, Shang-Wei Chen, Yu-Tai Shih, Sheng-Beng Hwang, Piotr Sitarek

**Affiliations:** 1 Department of Electronic Engineering, National Changhua University of Education, Changhua City 50074, Taiwan; 2 Department of Electronic Engineering, Ming Chi University of Technology, New Taipei City 243, Taiwan; 3 Department of Physics, National Changhua University of Education, Changhua City 500207, Taiwan; 4 Department of Electronic Engineering, Chienkuo Technology University, Changhua City 500020, Taiwan; 5 Department of Experimental Physics, Faculty of Fundamental Problems of Technology, Wrocław University of Science and Technology, 50370 Wrocław, Poland

**Keywords:** 2D semiconductors, absorption, van der Waals

## Abstract

In this study, we present the investigation of optical properties on a series of HfS_2−x_Se_x_ crystals with different Se compositions x changing from 0 to 2. We used the chemical-vapor transport method to grow these layered ternary compound semiconductors in bulk form. Their lattice constants and crystal properties were characterized by X-ray diffraction, high-resolution transmission electron microscopy, and Raman spectroscopy. We have performed absorption spectroscopies to determine their optical band-gap energies, which started from 2.012 eV with x = 0, and gradually shifts to 1.219 eV for x = 2. Furthermore, we measured the absorption spectroscopies at different temperatures in the range of 20–300 K to identify the temperature dependence of band-gap energies. The band-gap energies of HfS_2−x_Se_x_ were determined from the linear extrapolation method. We have noticed that the band-gap energy may be continuously tuned to the required energy by manipulating the ratio of S and Se. The parameters that describe the temperature influence on the band-gap energy are evaluated and discussed.

## 1. Introduction

There is a great need for high-speed electronics with low power consummation beyond the silicon-based devices. New flexible materials for electronics and optoelectronics applications with an atomic scale thickness have resulted in much exploration [1,2,3]. Novel two dimensional (2D) materials have attracted significant interest because of their unique fundamental properties [4,5]. Layered 2D transition metal dichalcogenides (TMDs) materials with the chemical formula MX_2_ (here M represents the transition metal and X represents a chalcogen) are considered to be candidates for next-generation electronic circuit applications. 2D TMD is stacked layer by layer in the form of X-M-X, consisting of two hexagonal planes of chalcogen elements [6]. The chalcogen layer-by-layer structure by van der Waals (vdW) interacts to form the bulk material, which allows for atomic control of its ultrathin thickness [7,8]. Unlike graphene without a band gap, the 2D TMDs with semiconductor behaviors can be adopted to the integration of electronic devices [9]. Up to now, 2D MX_2_ (M = Mo or W and X = S or Se) of single- or multi-layered TMDs with ultrathin thickness have been research topics of the fields of material science and nanotechnology [10]. Due to their specific optical and physical properties, the investigations of various device structures have included the heterostructure p-n junction diode [11], field effect transistor [12,13], fin-shaped field effect transistor [14,15], phototransistor [16], energy storage and conversion [17,18], sensors [19,20], biomedicine [21,22,23], and water remediation [24,25].

Among these TMDs materials, only a few studies on HfX_2_ (X = S or Se) compounds have been achieved [26]. The HfX_2_-based TMDs have high work function [27] and high mobility [28], allowing for their potential applications in nanoelectronic and optoelectronic devices [29,30,31,32]. The thermoelectric performance of HfS_2_ is also investigated [33]. The heterostructure of graphene/HfS_2_ as an electrode material for alkali-ion batteries is also performed [34]. However, the band gap of HfS_2_ and HfSe_2_ is fixed with no flexibility, which limits its ability in applications in devices. The bad gap of mixed ternary alloys can be tuned by adjusting the composition ratio, which allows for a wider detecting range in optoelectronic device applications. Due to the wide range of possible applications, the detailed temperature dependence of band-gap transitions is still unclear. Hence, more studies on the HfS_2−x_Se_x_ alloys concentrated on the temperature dependence of band-gap transitions are essential to pave the way for next-generation device applications.

In this study, the investigation of the structural properties of HfS_2−x_Se_x_ alloys measured by X-ray diffraction (XRD), high-resolution transmission electron microscopy (HRTEM), and Raman spectroscopy has been presented. We also characterized their compositions by electron probe X-ray micro-analyzer (EPMA). The band-gap energy determination of HfS_2−x_Se_x_ mixed crystals was carried out by the absorption spectroscopy. The parameters corresponding to the temperature dependence of the band-gap energies were also evaluated and discussed.

## 2. Experimental

Layered HfS_2−x_Se_x_ single crystals in bulk form were grown using the chemical-vapor transport method. I_2_ (~3.63 mg/cm^3^) was used as a transport agent for chemical transportation. A quartz ampoule with the size of 30 mm OD × 25 mm ID × 28 cm was used to contain the high-purity (Hf: 99.99%, S: 99.99%, and Se: 99.99%) constituent elements, which were weighted to fit the atomic ratio HfS_2−x_Se_x_ (0 ≤ x ≤ 2). A high-vacuum diffusion pump was used to maintained the quartz ampoule pressure at approximately 2 × 10^−5^ torr during the sealing process. Then, it was put into a two-zone furnace and two automatic temperature controllers were used to control the heater to hold the high temperature zone at 800 °C, and the low temperature zone at 650 °C in a duration of 14 days. The crystals thus grown have a surface area up to 10~20 mm^2^ and are 20–100 µm thick. In this work, the nominal composition x of six HfS_2−x_Se_x_ crystals varied as 0, 0.4, 0.8, 1.2, 1.6, and 2. The lattice structure and alloy compositions for all the HfS_2−x_Se_x_ crystals were checked by XRD and EPMA, respectively. The measured composition of the HfS_2−x_Se_x_ alloys by EPMA is listed in Table 1. The Raman spectroscopy has been performed on 3D Nanometer Scale Raman spectrometer (Tokyo Instruments, Nanofinder 30) with 488 nm laser. The laser power was set at about 1 mW to avoid the heating damages. The HRTEM images and selected area electron diffraction (SAED) patterns were taken by PHILIPS CM-200 TWIN FE-TEM to characterize the lattice structure of HfS_2−x_Se_x_ crystals.

The optical properties of the HfS_2−x_Se_x_ layered single crystals were studied by absorption measurements in the temperature range of 20–300 K. The light source with a 150 W quartz-halogen lamp was adopted to a PTI 0.25 m grating monochromator, focused on the sample with near-normal light incidence and collected by a silicon photodetector. The temperature-dependent measurements were carried out by a closed-cycle cryogenic refrigerator equipped with a digital thermometer controller with temperature stability in 0.5 K.

## 3. Results and Discussion

**Figure 1:**
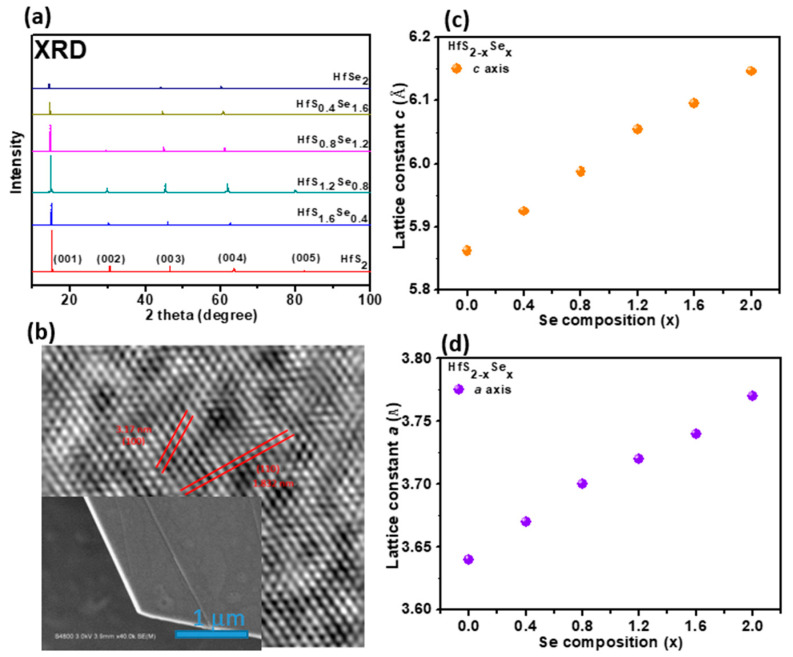
(**a**) XRD patterns of HfS_2−x_Se_x_ layered crystals with varying Se compositions. (**b**) Lattice constant *c* values deduced from XRD diffraction. (**c**) HRTEM image and SAED pattern of HfS_1.6_Se_0.4_ crystal. (**d**) The deduced lattice constants *a* of HfS_2−x_Se_x_ crystals from HRTEM.

The XRD patterns of HfS_2−x_Se_x_ layered single crystals with various Se content are depicted in Figure 1a. The major diffraction peaks of HfS_2−x_Se_x_ crystals fit in the figure of the CdI_2_ type hexagonal unit cell with the Pbnm space group. The strongest diffraction peak is assigned to the (001) plane; other diffraction peaks are assigned to the (002), (003), (004), and (005) planes [35]. The main peak position of the HfS_2−x_Se_x_ layered single crystals shifts to a lower angle gradually with increasing Se. The lattice constant c of HfS_2−x_Se_x_ crystals can be deduced according to Bragg’s law [36]. All of the XRD peaks, which are indicated as (001) to (005) from left to right, are derived from different planes stacking perpendicularly to the *c*-axis. Because all the XRD peaks indicate the same orientation, this result confirms the single crystalline nature of our HfS_2−x_Se_x_ crystal. In Equation (1) with the first-order approximation *n* = 1, and for the (001) orientation XRD peak located at 2θ ≈ 15° for the HfS_2_, we can find that d_001_ = 0.5862 nm. The lattice constant c can be deduced by substituting (hlk) = (001) into the Equation (2); we can see that the *c* value is equal to the value of d_001_, which is 0.5862 nm for HfS_2_ and gradually increases to 0.6146 nm for HfSe_2_.

2*d*sinθ = *n λ* (1)

(2) $$\frac{1}{d^{2}{}_{hkl}}=\frac{4}{3}\left(\frac{h^{2}+hk+k^{2}}{a^{2}}\right)+\frac{l^{2}}{c^{2}}$$

Figure 1b indicates the lattice constant *c* values deduced from XRD diffraction. The *c* value is 0.5862 nm for HfS_2_ and gradually increases to 0.6146 nm for HfSe_2_. The HfS_2−x_Se_x_ crystal structure was also characterized by HRTEM. Figure 1c shows the HRTEM image of HfS_1.6_Se_0.4_ crystal. The deduced lattice constants *a* of HfS_2−x_Se_x_ crystals from HRTEM are plotted in Figure 1d. The above results indicate that the crystal stoichiometry is in agreement with the nominal value.

**Figure 2:**
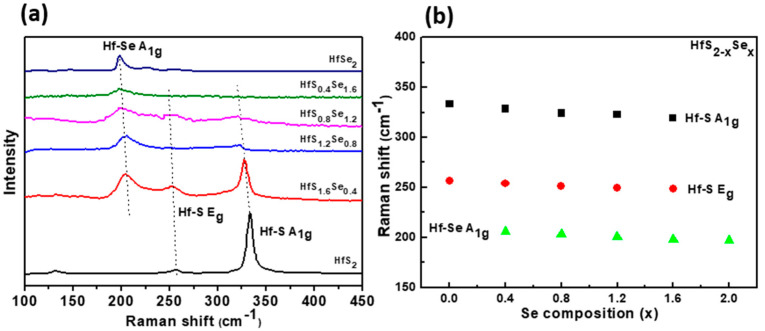
(**a**) Composition-dependent Raman spectra of HfS_2−x_Se_x_ layered crystals. (**b**) Evolution of Raman peak positions of HfS_2−x_Se_x_.

Figure 2a shows the characterization results of HfS_2−x_Se_x_ layered single crystals probed by Raman spectroscopy. The composition-dependent vibration modes of HfS_2−x_Se_x_ layered single crystals were assigned as A_1g_ (Hf-Se), E_g_ (Hf-S), and A_1g_ (Hf-S). The intensity of Hf-S related Raman modes decreases with increasing Se composition, and the vibration modes of Hf-Se are enhanced gradually. In Figure 2b, the Raman vibration modes of the HfS_2−x_Se_x_ layered single crystals are statistically depicted. The Raman frequencies of the A_1g_ and E_g_ vibration modes with increasing Se composition show a red-shift behavior. The Raman frequency shifts and intensity evolution with the S/Se atomic ratio are consistent with the reported literature [37] (HfS_2−x_Se_x_ alloys). The XRD measurements and Raman spectroscopy provide characterizations for the identification of the crystal structure and material phases of the HfS_2−x_Se_x_ layered single crystals.

**Figure 3:**
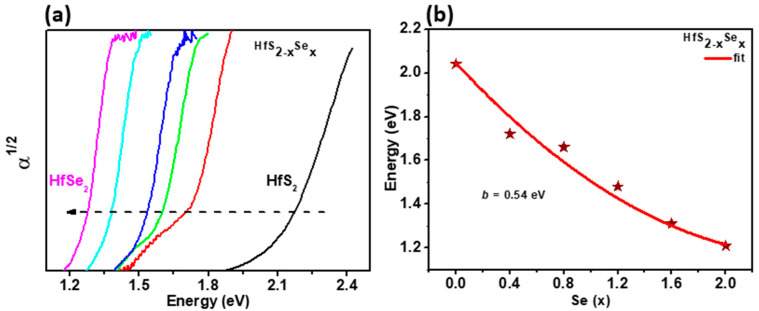
(**a**) The experimental absorption spectra of HfS_2−x_Se_x_ layered crystals at 300 K. (**b**) Composition-dependent band-gap energies for HfS_2−x_Se_x_ layered crystals at 300 K.

The room temperature absorption spectra of HfS_2−x_Se_x_ layered single crystals with various Se contents of x = 0, 0.4, 0.8, 1.2, 1.6, and 2, respectively, are shown in Figure 3a. We can observe that the absorption edge gradually shifts to a lower energy side with increasing Se content. The atomic number of Se is larger than S, which causes the band gap decrease with increasing Se content in HfS_2−x_Se_x_ layered single crystals. The band-gap energies (E_g_) for HfS_2−x_Se_x_ layered single crystals with different Se content were determined from the extrapolation of the absorption coefficient to the base line. The obtained band-gap energy values of HfS_2−x_Se_x_ layered single crystals at 300 K are 2.012, 1.729, 1.664, 1.543, 1.349, and 1.219 eV for Se content of x = 0, 0.4, 0.8, 1.2, 1.6, and 2, respectively. Compared to the band gap determined by the theoretical study of the HfS_2−x_Se_x_ monolayer, the results in study are in reasonable agreement with the reported theoretical results [38]. The discrepancy might be due to the material thickness effects and the difference between the experimental and the theoretical model. The composition dependence of the band-gap energy describes the degree of nonlinearity that can be fitted by the expression E_g_(x) = (x/2)E_g_(HfSe_2_) + (1 − (x/2))E_g_(HfS_2_) − *b*x/2(1 − (x/2)) [36], where E_g_ is the band-gap energy and *b* is the bowing parameter. In Figure 3b, the solid line represents the fit of experimental data with above equation. In this work, *b* is determined to be 0.54 eV, which is comparable with previous results of 0.68 eV for SnSSe [39] and 0.456 eV for ZnSSe [40] alloys. The deduced bowing parameters in this work are smaller than that the values 0.74–1.84 eV for the III-nitrides semiconductors. This might be due to the fact that the bowing parameter is dominated by various physical effects, structural and chemical disorder, atomic mismatch, strain, and carrier concentration [41].

**Figure 4:**
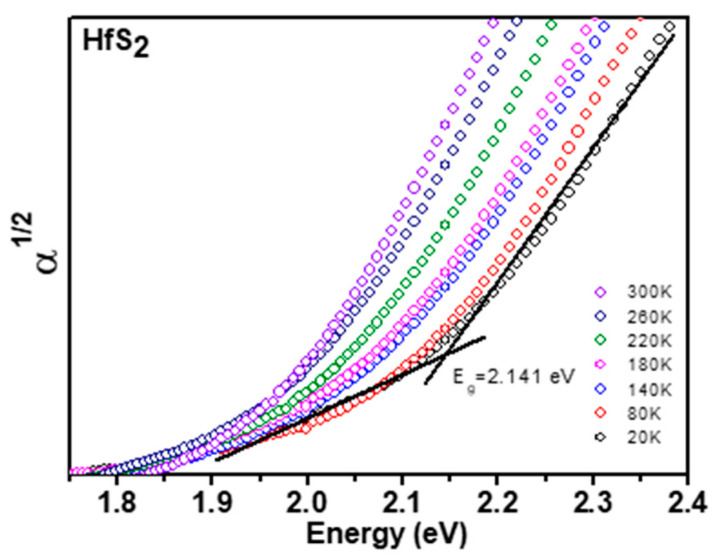
Experimental absorption spectra at various temperatures of the HfS_2_ layered crystals.

**Figure 5:**
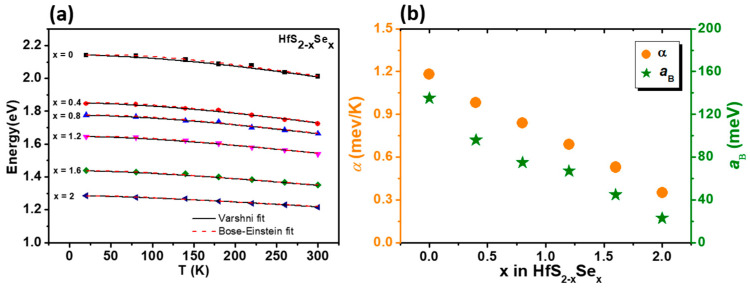
(**a**) The temperature dependence of the band-gap energies of HfS_2−x_Se_x_ layered crystals. The solid curves are least-squares fits to Equation (3), and the dashed curves are least-squares fits to Equation (4). (**b**) The parameters that describe the electron–phonon coupling of HfS_2−x_Se_x_ layered crystals are also plotted.

Displayed by dotted curves in Figure 4 are the experimental absorption spectra of the HfS_2_ layered single crystals at several temperatures between 20 and 300 K. There are a number of methods for bandgap calculation using absorption data, such as Tauc’s method, by taking α^1/2^ (indirect bandgap) or α^2^ (direct bandgap) methods. The relation derived by Tauc et al. [42] was intended for use with amorphous materials. They assume that the amorphous materials contain a localization of energy states; thus, absorption transitions do not need to conserve momentum. For single crystals with indirect energy gaps, the electron transition from the valence band to the conduction band, which is also understood as absorption transition, can be described by Fermi’s golden rule. The density states on both the valence and conduction bands will be included with phonon interactions to follow the rule of momentum conservation. The square root of the absorption coefficient plot could be available to extract the indirect band-gap energy [43]. As a general characteristic of most semiconductors, when the measurement temperature gradually rises, the band-gap energy exhibits a red-shift. The temperature variation of the band-gap energies obtained from the absorption measurements for the HfS_2−x_Se_x_ ternary-layered single crystals are plotted in Figure 5. The temperature-dependent evolution of the band-gap energy of the HfS_2−x_Se_x_ ternary-layered single crystals can be described by the Varshni semi-empirical model [44]:

E_g_(*T*) = E_g_(0) − α*T*^2^/(β + *T*) (3)

where E_g_(0) is the band-gap energies at 0 K. The constant α is related to the electron (exciton)–average phonon interaction strength, and β is closely related to the Debye temperature. The solid curves are least-squares fits to the Varshni semi-empirical model. Obtained from the fitting procedure, the Varshni parameters for the investigated crystals and, for comparison, other layered crystals such as MoSSe, WSSe 2D-layered semiconductors [45], III–V group semiconductors such as GaAs [46] and InP [47], II–VI group semiconductors such as ZnSe [48], and IV group semiconductors such as Si [46] are listed in Table 2.

The temperature dependence of band-gap energies can also be described by an expression containing the Bose–Einstein occupation factor for phonons [49,50]:

E_g_ (*T*) = E_g_ (0) − 2*a*_B_/[exp(Θ_B_ /T) − 1] (4)

where E_g_(0) is the band-gap energy at 0 K, *a*_B_ represents the strength of the electron (exciton)–average phonon interaction, and Θ_B_ corresponds to the average phonon temperature. In solid crystals, phonons play an important role in many physical properties. The variation of band-gap energy with temperature is majorly affected by lattice dilation and the electron–phonon interaction, which can be correlated with the phonon interaction [51,52].

The results obtained from fitting the parameter values according to the Bose–Einstein expression are listed in Table 2 as well.

The electron–phonon interaction strength parameter *α* from Varshni model can be easily related to electron–phonon interaction constant *a*_B_ and average phonon temperature Θ_B_ from Bose–Einstein expression by taking the high-temperature limit of both expressions. This yields *α* = 2*a*_B_/Θ_B_. Obtained from the fitting procedure to the experimental data with both expressions, the parameters presented in Table 2 show that this relation is matched. From Equation (4), it is straightforward to show that the high temperature limit of the slope of *E*(*T*) vs. the *T* curve approaches a value of −2*a*_B_/Θ_B_. The calculated value of −2*a*_B_/Θ_B_ for band-gap transition energies equals 0.78, −0.66, −0.58, −0.53, −0.42, and −0.28 meV/K for Se contents (x) = 0, 0.4, 0.8, 1.2, 1.6, and 2, respectively, which agrees well with the value of [d*E*/d*T*] = 0.75, −0.66, −0.50, −0.49, −0.43, and −0.32 meV/K, as obtained from the linear extrapolation of the high-temperature (140–300 K) absorption experimental data. It is noticed here that the temperature-dependence parameters indicate enhanced electron–phonon coupling with increasing sulfur composition. This may be due to the fact that the mass of S is lighter than that of Se Figure S1.

## 4. Conclusions

In conclusion, a series of HfS_2−x_Se_x_ ternary-layered single crystals were successfully grown by the chemical vapor transport method. The crystal phase properties of HfS_2−x_Se_x_ ternary-layered single crystals were characterized by XRD spectra and Raman spectra. The temperature dependence of the band-gap energy of HfS_2−x_Se_x_ ternary-layered single crystals was investigated by absorption spectroscopy in the temperature range between 20 and 300 K. Experimental data were fitted with the semi-empirical Varshni and Bose–Einstein expression. An enhanced electron–phonon coupling with higher sulfur content was observed by analyzing the parameters that describe the temperature dependence of the band-gap energies.

## Figures and Tables

**Table 1:** The measured composition (%) of the HfS_2−x_Se_x_ alloys assessed by EPMA.

HfS_2−x_Se_x_	Hf	S	Se
HfS_2_	34.25	65.74	
HfS_1.6_Se_0.4_	34.32	53.73	10.93
HfS_1.2_Se_0.8_	37.50	42.44	20.04
HfS_0.8_Se_1.2_	34.32	28.39	37.28
HfS_0.4_Se_1.6_	34.83	16.75	48.41
HfSe_2_	35.87		64.12

**Table 2:** _x_Se_x_ ternary-layered single crystals. The parameters of MoSSe, WSSe [45], GaAs [46], InP [47], ZnSe [48], and Si [46] are included for comparison.

Materials	Feature	*E*(0)	*α*	*β*	*a_B_*	Θ*_B_*	d*E*/d*T*
		(eV)	(m eV/K)	(K)	(m eV)	(K)	(m eV/K)
HfS_2_ ^a^	*E*_g_	2.141	1.18	496	135	342	−0.75
HfS_1.6_Se_0.4_ ^a^		1.849	0.98	425	96	288	−0.66
HfS_1.2_Se_0.8_ ^a^		1.775	0.84	367	75	258	−0.50
HfS_0.8_Se_1.2_ ^a^		1.646	0.69	300	67	250	−0.49
HfS_0.4_Se_1.6_ ^a^		1.436	0.54	247	45	214	−0.43
HfSe_2_ ^a^		1.284	0.35	169	23	161	−0.32
MoSSe ^b^		1.74	0.395	216			
WSSe ^b^		1.87	0.59	260			
GaAs ^c^	*E*_g_	1.517	0.55	225	57	240	
InP ^d^	*E*_g_	1.424	1.02	823	35.9	204	
ZnSe ^e^	*E*_g_	2.800	0.73	295	73	260	
Si ^f^	*E*_g_	1.170	4.73	636			

^b^ Ref. [45]; ^c^ Ref. [46]; ^d^ Ref. [47]; ^e^ Ref. [48]; and ^f^ Ref. [46].

## Data Availability

The data presented in this study are available in this article.

## Supplementary Materials

The following supporting information can be downloaded at: https://www.mdpi.com/article/10.3390/ma15186304/s1, Figure S1: Temperature−dependent absorption spectra of HfS_2−x_Se_x_.

## Sample Availability

The samples of the compounds are not available from the authors.
