# Supplementary figures and images for: Temperature-Dependent Absorption of Ternary HfS2−xSex 2D Layered Semiconductors
Source: Materials (Basel). 2022 Sep 11;15(18):6304. doi: 10.3390/ma15186304 (PMC9502516; doi:10.3390/ma15186304)

**Figure S1.** Temperature-dependent absorption spectra of  $\text{HfS}_{2-x}\text{Se}_x$  (a–f).

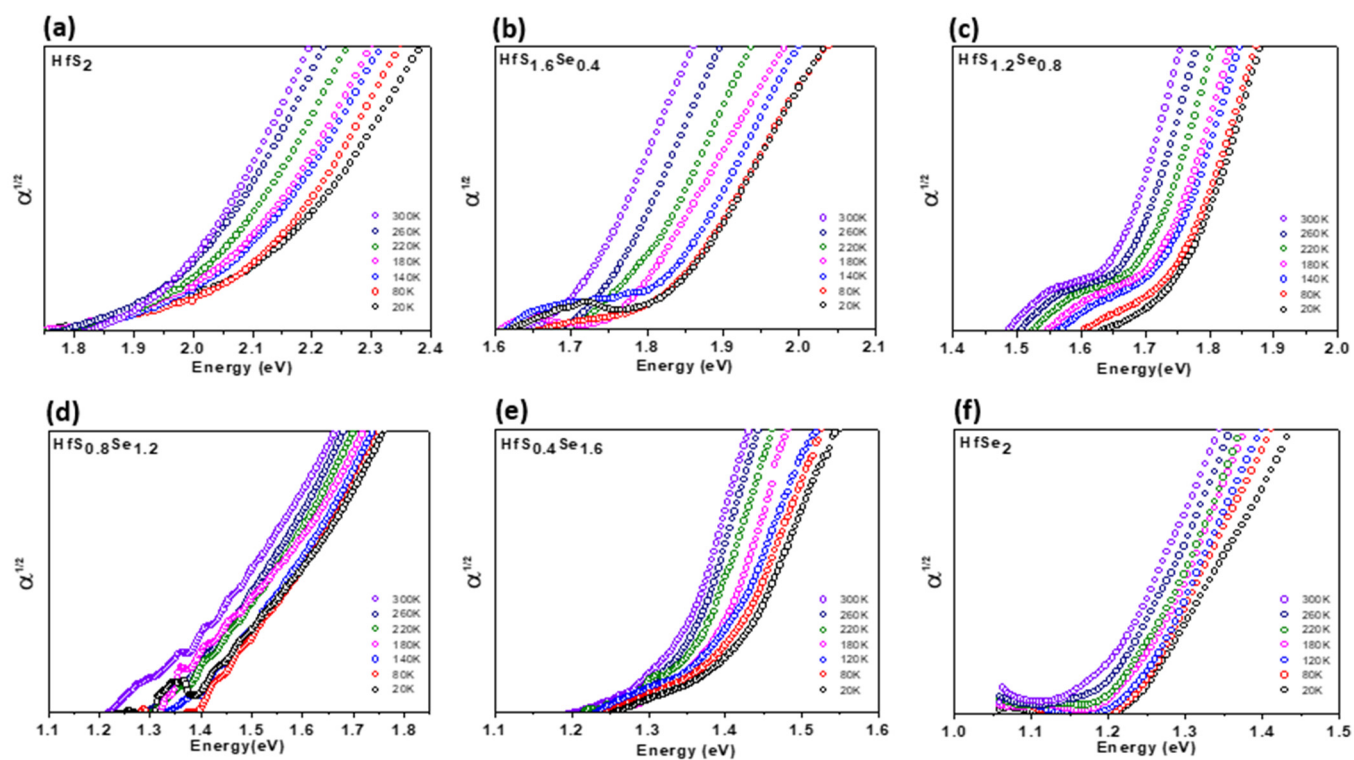

Supplement: Supplementary file 1 [file materials-15-06304-s001.zip › materials-1877618-supplementary.pdf]
